# Welche chronischen Erkrankungen kommen für eine fachärztliche Steuerung im Primärarztsystem infrage? Explorative Analyse anhand vertragsärztlicher Routinedaten

**DOI:** 10.1007/s00103-026-04293-9

**Published:** 2026-09-08

**Authors:** Sandra Mangiapane, Emil Hu, Dominik von Stillfried

**Affiliations:** Vorstandsbereich, Zentralinstitut für die Kassenärztliche Versorgung (Zi), Salzufer 8, 10587 Berlin, Deutschland

**Keywords:** Hausarztsystem, Primärversorgungssystem, Chronische Erkrankungen, Hausarzt, Facharzt, GP gate keeping, Primary care, Chronic diseases, General practitioner, Specialist

## Abstract

**Einleitung:**

Im geplanten Primärarztsystem sollen bei schweren chronischen Erkrankungen auch Fachärzte primärärztliche Aufgaben übernehmen können. Ziel der Arbeit war es, ein datenbasiertes Verfahren zur Identifikation potenziell geeigneter Erkrankungen zu entwickeln und die Versorgungsrelevanz einzelner Fachrichtungen darzustellen.

**Methoden:**

Auf Grundlage bundesweiter vertragsärztlicher Abrechnungsdaten für 2024 wurden für jeden ICD-3-Steller (erste 3 Stellen der International Classification of Diseases) die haus- und fachärztlichen Anteile an den Behandlungsfällen und Behandlungstagen je Patient bestimmt. Als Indikationen mit hoher fachärztlicher Versorgungsrelevanz galten Kategorien, bei denen bei mehr als der Hälfte der Patienten mindestens 60 % der Behandlungsfälle und mindestens 50 % der Behandlungstage auf Fachärzte entfielen. Exemplarisch wurden F33, G35, K51, M05 und N25 vertieft analysiert.

**Ergebnisse:**

173 ICD-3-Steller hatten eine hohe fachärztliche Versorgungsrelevanz. Bezogen auf die ausgewählten ICD-3-Steller hatten Patienten mit geschädigter Nierenfunktion (N25) den höchsten fachärztlichen Versorgungsanteil: 83 % der Patienten waren in nephrologischer Behandlung, auf Nephrologen entfielen 77 % der krankheitsspezifischen Fälle, aber nur 37 % aller Behandlungstage. Die höchsten Anteile an allen Behandlungstagen entfielen bei allen 5 Indikationen auf Hausärzte (43–55 %). 92–97 % dieser Patienten wurden hausärztlich versorgt.

**Diskussion:**

Sollten Fachärzte für diese Patienten die primärärztliche Steuerung übernehmen, müssten sie die Versorgung über ein breites Fachspektrum hinweg koordinieren. Indikationsübergreifende Steuerungsaufgaben dürften daher auf Einzelfälle oder auf das eigene Fachgebiet begrenzt bleiben.

## Abstract (en)

**Background:**

With the introduction of general practitioner (GP) gate keeping, specialists would also be able to assume primary care responsibilities for patients with severe chronic conditions. The aim of this study was to develop a data-driven method for identifying potentially suitable conditions and to illustrate the relevance of individual medical specialties to patient care.

**Methods:**

Using nationwide claims data of statutory health insurance physicians for 2024, the proportions of cases and treatment days per patient treated by primary care physicians and specialists were determined for each ICD‑3 code (the first three digits of the International Classification of Diseases). Categories were considered to have high relevance for specialist care if for more than half of the patients, at least 60% of treatment cases and at least 50% of treatment days were attributable to specialists. F33, G35, K51, M05, and N25 were analyzed in greater detail as examples.

**Results:**

There were 173 ICD-3 codes highly relevant to specialist care. With regard to the five selected ICD-3 codes, patients with impaired renal function (N25) had the highest proportion of specialist care: 83% of patients were receiving nephrological treatment, with nephrologists accounting for 77% of disease-specific cases but only 37% of all treatment days. For all five indications, general practitioners accounted for the highest proportion of all treatment days (43% to 55%). Of these patients, 92 to 97% were treated by general practitioners.

**Discussion:**

If specialists were to take on the role of primary care coordinators for these patients, they would have to coordinate care across a broad range of medical specialties. This would likely limit their cross-specialty coordination tasks to individual cases or restrict it to their own specialty.

## Einleitung

Die Bundesregierung beabsichtigt, bis 2029 ein verbindliches Primärarztsystem mit Haus- und Kinderärzten einzuführen. Ziele sind eine zielgerichtetere Versorgung und eine schnellere Terminvergabe. Der Zugang zur fachärztlichen Versorgung soll grundsätzlich einer Überweisung bedürfen. Ausnahmen sind für die Fachrichtungen Augenheilkunde und Gynäkologie vorgesehen. Für Patienten mit „spezifischen schweren chronischen Erkrankungen“ sollen zudem geeignete (alternative) Lösungen erarbeitet werden, beispielsweise in Form von Jahresüberweisungen oder der Festlegung eines Fachinternisten als steuernden Primärarzt im Einzelfall [1].

Bislang ist unklar, welche Erkrankungen als „spezifische schwere chronische Erkrankungen“ gelten und wer diese definiert. Bereits bestehende Disease-Management-Programme (DMP) für chronische Volkskrankheiten wie Diabetes mellitus oder koronare Herzkrankheit sehen den Hausarzt als steuernden Arzt vor [2]. Daher ist zu vermuten, dass die geplante Ausnahmeregelung für andere, seltenere oder komplexere chronische Erkrankungen oder bestimmte ausgeprägte Behandlungsepisoden, wie etwa eine Tumorbehandlung, gelten könnte.

Ebenso offen ist, welche Aufgaben eine fachärztliche Bezugspraxis übernehmen soll: Beschränkt sich ihre Koordination auf die betreffende Erkrankung oder Behandlungsepisode oder umfasst sie auch Komorbiditäten und Grundleistungen wie Impfungen? Vermutlich wird dies in Abhängigkeit von den Versorgungsbedarfen unterschiedlicher Patientengruppen differenziert beurteilt werden müssen.

Analysen auf Basis von vertragsärztlichen und vertragspsychotherapeutischen Abrechnungsdaten des Jahres 2007 zeigen, dass lebensverändernde chronische Erkrankungen mit einer überdurchschnittlich hohen Anzahl an Arztkontakten einhergehen [3]. Während die durchschnittliche Anzahl an Arztkontakten pro Patient und Jahr über alle Patienten hinweg 17 beträgt, weisen Patienten mit chronischen Erkrankungen im Mittel 34 Arztkontakte pro Jahr auf. Die höchste Anzahl an Arztkontakten haben Patienten nach Organtransplantationen, Patienten mit Niereninsuffizienz, chronischer Hepatitis oder bösartigen Neubildungen. Bezüglich der Frage, welcher Anteil an Arztkontakten auf Hausärzte entfällt, konnte zudem eine deutliche Abhängigkeit vom Alter der Versicherten festgestellt werden. Während in der Altersgruppe der 20- bis 35-Jährigen ca. 46 % der Arztkontakte auf die Hausärzte entfallen, sind es bei den über 80-Jährigen 67 % bis 80 % [3].

Bisher wurde wenig untersucht, ob und in welchem Umfang die Praxen bestimmter Fachrichtungen zum maßgeblichen Bezugspunkt für die Patienten werden, sodass diese sich für die Rolle der primären Bezugspraxis in einem Primärarztsystem eignen würden. Ziel dieser Originalarbeit ist es, ein Verfahren vorzustellen, mit dem ICD(International Statistical Classification of Diseases and Related Health Problems)-3-Kategorien identifiziert werden können, die potenziell für eine fachärztliche Steuerung geeignet sind, und die Versorgungsrelevanz einzelner Fachrichtungen für ausgewählte Kategorien zu untersuchen.

## Methoden

### Datenquelle

Als Datenquelle wurden die pseudonymisierten, bundesweiten vertragsärztlichen und vertragspsychotherapeutischen Abrechnungsdaten des Jahres 2024 verwendet. Es wurden nur Behandlungsfälle von gesetzlich krankenversicherten Patienten eingeschlossen, die im Jahr 2024 mindestens 18 Jahre alt waren. Behandlungsfälle, die ausschließlich von Laborärzten, Pathologen oder Fachwissenschaftlern abgerechnet wurden, wurden von der Analyse ausgeschlossen. Ein Behandlungsfall umfasst die Behandlung eines Patienten durch dieselbe Arztpraxis innerhalb eines Kalendervierteljahres zulasten derselben Krankenkasse. Selektivvertragsärztliche Fälle konnten nicht berücksichtigt werden.

### Zuteilung der Patienten zu ICD-3-Kategorien

Für jeden Patienten wurden die im Jahr 2024 als gesichert codierten Diagnosen den 3‑stelligen Kategorien der International Classification of Diseases in der Version ICD-10-GM (ICD-3-Steller) zugeordnet. Diese Verdichtung erfolgte aus Gründen der Übersichtlichkeit. Dabei konnten die Patienten mehreren ICD-3-Stellern zugeordnet werden. Für jeden ICD-3-Steller wurden folgende Kennzahlen bestimmt: die Zahl der Patienten, Mittelwert und Median der Behandlungsfälle und Behandlungstage je Patient sowie die fachärztlichen Anteile an allen Behandlungsfällen und Behandlungstagen je Patient. Unter Behandlungstagen ist die Anzahl an Tagen zu verstehen, an denen für einen Patienten mindestens eine Gebührenordnungsposition (GOP) des Einheitlichen Bewertungsmaßstabes (EBM) abgerechnet wurde. Tage, an denen zwar ein Arzt-Patienten-Kontakt stattfand, die aber beispielsweise aufgrund einer zuvor im Quartal bereits abgerechneten Quartalspauschale nicht mit der Abrechnung einer weiteren GOP einhergehen, können dabei nicht erfasst werden, sodass die Behandlungstage nur als Annäherung für die Anzahl an tatsächlich stattgefundenen Arzt-Patienten-Kontakten betrachtet werden können. Darüber hinaus können die Behandlungstage in den Behandlungsfällen, in denen Diagnosen aus mehreren ICD-3-Kategorien codiert wurden oder an denen mehrere Fachgruppen beteiligt waren, nicht konkret einem ICD-3-Code beziehungsweise einer Fachgruppe zugeordnet werden. In diesen Fällen wurden die Behandlungstage allen vorkommenden ICD-3-Codes und allen beteiligten Fachgruppen zugeordnet.

Aus Gründen der Übersichtlichkeit wurden für die Auswertung nur ICD-3-Steller mit mindestens 1000 betroffenen Patienten berücksichtigt.

*Auswahl potenziell geeigneter ICD-3-Steller: *Ausgehend von der Überlegung, dass Erkrankungen, die für eine fachärztliche Steuerung infrage kommen, bereits aktuell zu einem Großteil fachärztlich betreut werden, wurden in einem nächsten Schritt die ICD-3-Steller mit einem medianen Anteil fachärztlicher Behandlungsfälle an allen Behandlungsfällen von mindestens 60 % und einem medianen Anteil fachärztlicher Behandlungstage an allen Behandlungstagen von mindestens 50 % ermittelt. Diese Schwellenwerte wurden ausgehend von der jeweils zugrunde liegenden Verteilung des Anteils fachärztlicher Behandlungsfälle an allen Behandlungsfällen pro Patient und des Anteils fachärztlicher Behandlungstage an allen Behandlungstagen pro Patient festgelegt. Sie wurden jeweils so gewählt, dass sie deutlich über dem jeweiligen Median lagen (Tab. 1).

**Tab. 1:** Anzahl an Behandlungsfällen und Behandlungstagen pro GKV-Versicherten im Jahr 2024 (ab 18 Jahren, ohne Behandlungsfälle, die ausschließlich von Laborärzten, Pathologen oder Fachwissenschaftlern abgerechnet wurden)

Anzahl Patienten	59.717.743
Behandlungsfälle pro Patient: durchschnittliche Anzahl (Median)	8 (6)
Behandlungstage pro Patient: durchschnittliche Anzahl (Median)	14 (10)
Hausärztliche Behandlungsfälle pro Patient: durchschnittliche Anzahl (Median)	4 (4)
Hausärztliche Behandlungstage pro Patient: durchschnittliche Anzahl (Median)	9 (6)
Fachärztliche Behandlungsfälle pro Patient: durchschnittliche Anzahl (Median)	5 (4)
Fachärztliche Behandlungstage pro Patient durchschnittliche Anzahl (Median)	9 (5)
Anteil fachärztlicher Behandlungstage pro Patient: durchschnittlicher Anteil (Median)	50 % (50 %)
Anteil fachärztlicher Behandlungstage pro Patient: durchschnittlicher Anteil (Median)	45 % (44 %)

Eigene Berechnungen auf Basis vertragsärztlicher und vertragspsychotherapeutischer Abrechnungsdaten 2024

ICD-3-Steller mit Bezug zu gynäkologischen Erkrankungen, Erkrankungen im Zusammenhang mit Schwangerschaft und Geburt sowie Augenerkrankungen wurden ausgeschlossen, da diese Fachrichtungen gemäß Koalitionsvertrag von der verpflichtenden Primärarztsteuerung ausgenommen werden sollen [1]. Ebenfalls ausgeschlossen wurden ICD-3-Steller mit einem eindeutigen Bezug zu Zahnerkrankungen oder zu akuten Erkrankungen, also Erkrankungen ohne erkennbar dauerhaften Betreuungsbedarf (zum Beispiel B97: Viren als Ursache von Krankheiten, die in anderen Kapiteln klassifiziert sind, oder J99: Krankheiten der Atemwege bei anderenorts klassifizierten Krankheiten), sowie „Kennzeichnungscodes“ (zum Beispiel Z46: Versorgen mit und Anpassen von anderen medizinischen Geräten oder Hilfsmitteln oder Z94: Zustand nach Organ- oder Gewebetransplantation). Aus den so ermittelten ICD-3-Stellern wurden anschließend exemplarisch 5 Kategorien ausgewählt, die jeweils eine bestimmte chronische Erkrankung mit relevanter Fallzahl abbilden. Dabei wurde darauf geachtet, unterschiedliche Fachgebiete einzubeziehen. Für diese ICD-3-Steller wurde analysiert, welche Fachrichtungen an der Versorgung der jeweiligen Patienten beteiligt waren und welcher Anteil der Behandlungsfälle und der Behandlungstage auf die jeweilige Fachrichtung entfiel. Pro ICD-3-Steller wurden die 10 Fachgruppen mit dem höchsten Anteil an den krankheitsspezifischen Behandlungstagen der Patienten ermittelt. Als krankheitsspezifische Behandlungstage wurden dabei solche definiert, die aus Behandlungsfällen stammten, in denen der jeweilige ICD-3-Steller codiert wurde.

## Ergebnisse

Insgesamt nahmen im Jahr 2024 rund 60 Mio. erwachsene gesetzlich Krankenversicherte mindestens einmal eine vertragsärztliche oder vertragspsychotherapeutische Leistung in Anspruch. Dies sind rund 97 % aller gesetzlich Krankenversicherten im Erwachsenenalter. Im Durchschnitt entfielen auf diese Patienten 8 Behandlungsfälle und 14 Behandlungstage pro Jahr. Dies entspricht durchschnittlich 1,75 Behandlungstagen je Behandlungsfall. Die Hälfte der Patienten hatte weniger als 6 Behandlungsfälle und weniger als 10 Behandlungstage pro Jahr (Tab. 1).

### Behandlungstage je ICD-3-Steller

Nach Ausschluss der 222 ICD-3-Steller mit weniger als 1000 betroffenen Patienten verteilten sich die Behandlungsfälle und Behandlungstage der Patienten über 1506 unterschiedliche ICD-3-Steller. Erwartungsgemäß zeigte sich bei ICD-3-Stellern mit Bezug zu chronischen Erkrankungen und längerfristigem Behandlungsbedarf die höchste mediane Anzahl an Behandlungstagen pro Patient. Die im Median höchste Gesamtzahl an Behandlungstagen über alle Behandlungsanlässe pro Patient (Tab. 2) zeigte sich bei Patienten mit Dialysebehandlung (Z49, 137 Behandlungstage pro Patient), bei Patienten mit psychischen und Verhaltensstörungen durch Opioide (F11, 45 Behandlungstage pro Patient) und bei Patienten mit Anämie (D63, 40 Behandlungstage pro Patient). Die niedrigste Anzahl an Behandlungstagen pro Patient entfiel auf Patienten mit den ICD-3-Stellern Y09-Tätlicher Angriff, N46-Sterilität beim Mann und K01-Retinierte und impaktierte Zähne (jeweils 10 Behandlungstage pro Patient).

**Tab. 2:** ICD-3-Steller mit der höchsten und der niedrigsten Anzahl an Behandlungstagen pro Patient (Median)

ICD‑3	Diagnose	Anzahl Patienten	Anzahl Behandlungstage pro Patient (Median)
Z49	Dialysebehandlung	122.711	137
F11	Psychische und Verhaltensstörungen durch Opioide	134.431	45
D63	Anämie bei chronischen, anderenorts klassifizierten Krankheiten (C00-D48+, N18.3-N18.5+)	202.703	40
N25	Krankheiten infolge Schädigung der tubulären Nierenfunktion	182.208	36
Z75	Probleme mit Bezug auf medizinische Betreuungsmöglichkeiten oder andere Gesundheitsversorgung	20.505	36
E43	Nicht näher bezeichnete erhebliche Energie- und Eiweißmangelernährung	6009	33
T86	Versagen und Abstoßung von transplantierten Organen und Geweben	15.657	33
Y82	Medizintechnische Geräte und Produkte im Zusammenhang mit Zwischenfällen bei diagnostischer und therapeutischer Anwendung	1648	33
C38	Bösartige Neubildung des Herzens, des Mediastinums und der Pleura	6694	32
C39	Bösartige Neubildung sonstiger und ungenau bezeichneter Lokalisationen des Atmungssystems und sonstiger intrathorakaler Organe	1579	32
L58	Radiodermatitis	8561	32
N01	Rapid-progressives nephritisches Syndrom	3128	32
U81	Gramnegative Erreger mit bestimmten Antibiotikaresistenzen, die besondere therapeutische oder hygienische Maßnahmen erfordern	17.669	32
C48	Bösartige Neubildung des Retroperitoneums und des Peritoneums	11.764	31
C77	Sekundäre und nicht näher bezeichnete bösartige Neubildung der Lymphknoten	183.044	31
C79	Sekundäre bösartige Neubildung an sonstigen und nicht näher bezeichneten Lokalisationen	255.420	31
C90	Plasmozytom und bösartige Plasmazellen-Neubildungen	60.682	31
C93	Monozytenleukämie	5017	31
T82	Komplikationen durch Prothesen, Implantate oder Transplantate im Herzen und in den Gefäßen	71.397	31
C66	Bösartige Neubildung des Ureters	8349	30
…	Aus Platzgründen wurden die 1466 ICD-3-Steller, die zwischen den 20 ICD-3-Stellern mit der höchsten Anzahl an Behandlungstagen pro Patient und den 20 ICD-3-Stellern mit dem niedrigsten Anteil an Behandlungstagen pro Patient liegen, nicht aufgelistet		
N86	Erosion und Ektropium der Cervix uteri	844.859	12
N94	Schmerz und andere Zustände im Zusammenhang mit den weiblichen Genitalorganen und dem Menstruationszyklus	2.601.981	12
R50	Fieber sonstiger und unbekannter Ursache	691.931	12
R62	Ausbleiben der erwarteten normalen physiologischen Entwicklung	11.813	12
T62	Toxische Wirkung sonstiger schädlicher Substanzen, die mit der Nahrung aufgenommen wurden	15.737	12
B01	Varizellen (Windpocken)	9973	11
B34	Viruskrankheit nicht näher bezeichneter Lokalisation	2.051.729	11
B80	Enterobiasis (Oxyuriasis)	90.197	11
B83	Sonstige Helminthosen	56.336	11
J00	Akute Rhinopharyngitis (Erkältungsschnupfen)	2.204.570	11
J03	Akute Tonsillitis	1.661.694	11
J06	Akute Infektionen an mehreren oder nicht näher bezeichneten Lokalisationen der oberen Atemwege	14.545.361	11
N44	Hodentorsion und Hydatidentorsion	5944	11
T15	Fremdkörper im äußeren Auge	139.917	11
T51	Toxische Wirkung von Alkohol	38.749	11
W87	Exposition gegenüber elektrischem Strom	1652	11
Z30	Kontrazeptive Maßnahmen	9.700.791	11
K01	Retinierte und impaktierte Zähne	44.281	10
N46	Sterilität beim Mann	117.230	10
Y09	Tätlicher Angriff	12.685	10

*Quelle*: eigene Berechnungen. *Datenquelle*: vertragsärztliche und vertragspsychotherapeutische Abrechnungsdaten 2024 (ohne Behandlungsfälle, die ausschließlich von Laborärzten, Pathologen oder Fachwissenschaftlern abgerechnet wurden), nur ICD-3-Steller mit mindestens 1000 Patienten

### Chronische Erkrankungen mit einem hohen fachärztlichen Behandlungsanteil

Nach Ausschluss von ICD-3-Stellern mit einem medianen Anteil fachärztlicher Behandlungsfälle an allen Behandlungsfällen von weniger als 60 % und einem medianen Anteil fachärztlicher Behandlungstage an allen Behandlungstagen von weniger als 50 % und weiterer für die Fragestellung nicht relevanter ICD-3-Codes (siehe Methoden) verblieben 173 ICD-3-Steller, die sich über die in Tab. 3 aufgeführten ICD-Kapitel verteilten.

**Tab. 3:** Verteilung der 173 ICD-3-Steller mit einem hohen fachärztlichen Behandlungsanteil* über die ICD-Kapitel

Kapitel		Gliederung	Anzahl ICD-3-Steller	Anzahl Patienten	Anzahl Behandlungsfälle	Anzahl Behandlungstage
1	Bestimmte infektiöse und parasitäre Krankheiten	A00-B99	Ausgeschlossen			
2	Neubildungen	C00-D48	32	13.066.240	35.516.609	80.937.849
3	Krankheiten des Blutes und der blutbildenden Organe sowie bestimmte Störungen mit Beteiligung des Immunsystems	D50-D90	2	378.857	1.086.270	9.966.578
4	Endokrine, Ernährungs- und Stoffwechselkrankheiten	E00-E90	5	1.802.894	4.858.527	15.283.176
5	Psychische und Verhaltensstörungen	F00-F99	17	16.433.625	53.157.807	147.166.147
6	Krankheiten des Nervensystems	G00-G99	12	6.670.654	20.761.127	52.756.012
7	Krankheiten des Auges und der Augenanhangsgebilde	H00-H59	Ausgeschlossen			
8	Krankheiten des Ohres und des Warzenfortsatzes	H60-H95	6	6.992.423	11.794.095	16.813.757
9	Krankheiten des Kreislaufsystems	I00-I99	3	550.935	850.457	1.650.718
10	Krankheiten des Atmungssystems	J00-J99	8	8.134.435	20.115.258	41.885.876
11	Krankheiten des Verdauungssystems	K00-K93	13	6.124.949	16.317.445	41.332.419
12	Krankheiten der Haut und der Unterhaut	L00-L99	23	12.535.521	26.594.821	57.430.941
13	Krankheiten des Muskel-Skelett-Systems und des Bindegewebes	M00-M99	37	28.667.102	97.781.709	235.781.983
14	Krankheiten des Urogenitalsystems	N00-N99	13	3.179.179	7.395.701	22.889.775
15	Schwangerschaft, Geburt und Wochenbett	O00-O99	Ausgeschlossen			
16	Bestimmte Zustände, die ihren Ursprung in der Perinatalperiode haben	P00-P96	Ausgeschlossen			
17	Angeborene Fehlbildungen, Deformitäten und Chromosomenanomalien	Q00-Q99	Ausgeschlossen			
18	Symptome und abnorme klinische und Laborbefunde, die anderenorts nicht klassifiziert sind	R00-R99	Ausgeschlossen			
19	Verletzungen, Vergiftungen und bestimmte andere Folgen äußerer Ursachen	S00-T98	Ausgeschlossen			
20	Äußere Ursachen von Morbidität und Mortalität	V01-Y84	Ausgeschlossen			
21	Faktoren, die den Gesundheitszustand beeinflussen und zur Inanspruchnahme des Gesundheitswesens führen	Z00-Z99	1	122.711	536.293	11.978.364
22	Schlüsselnummern für besondere Zwecke	U00-U99	1	363.712	930.174	2.533.229

Eigene Berechnungen. *Datenquelle*: vertragsärztliche und vertragspsychotherapeutische Abrechnungsdaten 2024 (ohne Behandlungsfälle, die ausschließlich von Laborärzten, Pathologen oder Fachwissenschaftlern abgerechnet wurden), nur Fälle von Patienten ab 18 Jahren und nur ICD-3-Steller mit mindestens 1000 Patienten

* Medianer Anteil fachärztlicher Behandlungsfälle an allen Behandlungsfällen weniger als 60 % und medianer Anteil fachärztlicher Behandlungstage an allen Behandlungstagen weniger als 50 %

ICD-3-Steller aus den Kapiteln 1, 18, 19, 20, 21, 22 wurden vorab entweder vollständig oder teilweise aufgrund der Kriterien „Kennzeichnungscode“ oder „akuter Anlass“ ausgeschlossen. ICD-3-Steller aus den Kapiteln 7, 15, 16 und 17 wurden aufgrund des Bezugs zu Schwangerschaft, Geburt oder augenärztlicher Versorgung vollständig herausgenommen. ICD-3-Steller aus den übrigen Kapiteln wurden hingegen hauptsächlich aufgrund eines niedrigeren fachärztlichen Behandlungsanteils ausgeschlossen (medianer Anteil fachärztlicher Behandlungsfälle an allen Behandlungsfällen weniger als 60 % und medianer Anteil fachärztlicher Behandlungstage an allen Behandlungstagen weniger als 50 %)

Die größten Patientenzahlen sowie Behandlungsfälle und -tage entfielen auf Kapitel 13 (Krankheiten des Muskel-Skelett-Systems und des Bindegewebes), Kapitel 5 (Psychische und Verhaltensstörungen) und Kapitel 2 (Neubildungen). Die wenigsten Patienten, Behandlungsfälle und Behandlungstage entfielen hingegen auf die Kapitel 9 (Krankheiten des Kreislaufsystems), 3 (Krankheiten des Blutes …) und 22 (Schlüsselnummern für besondere Zwecke), wobei hier ausschließlich der ICD-3-Steller U09 (Post-COVID-19-Zustand) enthalten war. Auf den einzigen im Kapitel 21 (Faktoren, die den Gesundheitszustand beeinflussen …) verbleibenden ICD-3-Steller Z49 (Dialysebehandlung) verteilten sich zwar die wenigsten Patienten, diese wiesen aber eine hohe Anzahl an Behandlungstagen auf.

ICD-3-Steller aus den Kapiteln 1 (Bestimmte infektiöse und parasitäre Krankheiten), 18 (Symptome und abnorme klinische und Laborbefunde, die anderenorts nicht klassifiziert sind), 19 (Verletzungen, Vergiftungen und bestimmte andere Folgen äußerer Ursachen), 20 (Äußere Ursachen von Morbidität und Mortalität), 21 (Faktoren, die den Gesundheitszustand beeinflussen und zur Inanspruchnahme des Gesundheitswesens führen), 22 (Schlüsselnummern für besondere Zwecke) wurden vorab entweder vollständig oder teilweise aufgrund der Kriterien „Kennzeichnungscode“ oder „akuter Anlass“ ausgeschlossen. ICD-3-Steller aus den Kapiteln 7 (Krankheiten des Auges und der Augenanhangsgebilde), 15 (Schwangerschaft, Geburt und Wochenbett), 16 (Bestimmte Zustände, die ihren Ursprung in der Perinatalperiode haben) und 17 (Angeborene Fehlbildungen, Deformitäten und Chromosomenanomalien) wurden aufgrund des Bezugs zu Schwangerschaft, Geburt oder augenärztlicher Versorgung vollständig herausgenommen. ICD-3-Steller aus den übrigen Kapiteln wurden hingegen hauptsächlich aufgrund eines niedrigeren fachärztlichen Behandlungsanteils ausgeschlossen (medianer Anteil fachärztlicher Behandlungsfälle an allen Behandlungsfällen weniger als 60 % und medianer Anteil fachärztlicher Behandlungstage an allen Behandlungstagen weniger als 50 %).

### Exemplarisch ausgewählte chronische Erkrankungen

Für eine genauere Betrachtung der an der Versorgung einer chronischen Erkrankung maßgeblich beteiligten Fachgruppen wurden die in Tab. 4 aufgeführten ICD-3-Steller ausgewählt (dargestellt sind jeweils die 10 Fachgruppen mit dem höchsten Anteil an den krankheitsspezifischen Behandlungstagen der Patienten).

**Tab. 4:** Ausgewählte ICD-3-Steller mit einem hohen fachärztlichen Versorgungsanteil* nach TOP-10-Fachgruppen (sortiert nach durchschnittlichem Anteil an Behandlungstagen mit ICD-3-Steller bei der jeweiligen Fachgruppe)

ICD‑3	Fachgruppe	Anteil Patienten gesamt bei der Fachgruppe	Anteil Patienten mit ICD‑3 bei der Fachgruppe	Durchschnittlicher Anteil der Behandlungstage bei der Fachgruppe an den Behandlungstagen eines Patienten	
				Fälle mit dem jeweiligen ICD-3-Code	Alle Fälle
*Spalte 1*	*Spalte 2*	*Spalte 3 (%)*	*Spalte 4 (%)*	*Spalte 5 (%)*	*Spalte 6 (%)*
F33-Rezidivierende depressive Störung (*N* = 3.382.274)	Hausärzte	96,1	60,3	88	50
	Nichtärztliche Psychotherapeuten	24,2	17,2	80	38
	Kinder- und Jugendpsychiatrie und -psychotherapie	0,3	0,1	79	28
	Ärztliche Psychotherapeuten	6,0	4,3	78	33
	Urologie	14,2	0,5	77	11
	Innere Medizin, Hämatologie/Onkologie	2,5	0,1	72	17
	Psychiatrie	24,3	18,5	72	23
	Sonstige Ärzte	12,7	6,1	70	16
	Nervenheilkunde	13,7	8,1	69	19
	Gynäkologie	37,7	1,3	68	13
G35-Multiple Sklerose (Encephalomyelitis disseminata) (*N* = 301.733)	Hausärzte	92,1	83,1	60	47
	Neurologie	55,9	52,0	42	29
	Nervenheilkunde	18,1	16,7	37	26
	Nichtärztliche Psychotherapeuten	6,2	0,6	36	29
	Sonstige Ärzte	14,0	10,4	36	21
	Ärztliche Psychotherapeuten	1,4	0,5	35	26
	Psychiatrie	6,0	4,2	31	20
	Kinder- und Jugendmedizin	0,5	0,1	30	11
	Innere Medizin, Nephrologie	1,5	0,8	27	16
	Innere Medizin, FA ohne Schwerpunkt	3,4	1,5	27	14
K51-Colitis ulcerosa (*N* = 364.809)	Hausärzte	95,9	84,2	82	54
	Innere Medizin, Gastroenterologie	39,6	32,5	50	27
	Kinder- und Jugendmedizin	0,8	0,2	49	17
	Ärztliche Psychotherapeuten	1,3	0,2	48	27
	Innere Medizin, FA ohne Schwerpunkt	10,2	6,8	47	22
	Nichtärztliche Psychotherapeuten	4,9	0,2	46	30
	Chirurgie	18,0	3,8	44	13
	Innere Medizin, schwerpunktübergreifend	1,5	0,8	44	19
	Sonstige Ärzte	7,1	1,9	43	15
	Innere Medizin, Hämatologie/Onkologie	3,3	1,0	42	21
M05-Seropositive chronische Polyarthritis (*N* = 301.159)	Hausärzte	96,0	50,0	88	52
	Innere Medizin, Rheumatologie	60,0	50,2	79	22
	Augenheilkunde	44,2	0,9	78	9
	Orthopädie	45,9	14,4	76	15
	Gynäkologie	35,0	0,6	69	10
	Urologie	15,5	0,2	69	11
	Ärztliche Psychotherapeuten	0,9	0,05	68	21
	Hautärzte	26,3	0,3	67	8
	Physikalische und rehabilitative Medizin	3,1	1,1	67	13
	Psychiatrie	3,8	0,1	65	14
*N*25-Krankheiten infolge Schädigung der tubulären Nierenfunktion (*N* = 182.208)	Innere Medizin, Nephrologie	82,8	77,0	92	37
	Gynäkologie	13,9	0,4	92	8
	Sonstige Leistungserbringer	38,3	12,1	84	21
	Nuklearmedizin	5,7	0,1	81	4
	Urologie	27,0	0,8	79	10
	Innere Medizin, schwerpunktübergreifend	3,8	2,1	78	24
	Innere Medizin, Rheumatologie	2,5	0,3	78	14
	Orthopädie	26,8	0,5	78	9
	Innere Medizin, Endokrinologie	1,8	0,4	77	14
	Hausärzte	92,8	23,1	76	43

Eigene Berechnungen. *Datenquelle*: vertragsärztliche und vertragspsychotherapeutische Abrechnungsdaten 2024 (ohne Behandlungsfälle, die ausschließlich von Laborärzten, Pathologen oder Fachwissenschaftlern abgerechnet wurden), nur Fälle von Patienten ab 18 Jahren und nur ICD-3-Steller mit mindestens 1000 Patienten

* Medianer Anteil fachärztlicher Behandlungsfälle an allen Behandlungsfällen weniger als 60 % und medianer Anteil fachärztlicher Behandlungstage an allen Behandlungstagen weniger als 50 %

Je nach Erkrankung waren 92–97 % der Patienten im Jahr 2024 in hausärztlicher Versorgung (Tab. 4, Spalte 3), wobei auf Hausärzte 43–55 % aller Behandlungstage (Spalte 6) entfielen.

Die Bedeutung einzelner fachärztlicher Fachgruppen fällt mit Blick auf die durchschnittlichen Anteile an krankheitsspezifischen Fällen (Spalte 4) und Behandlungstagen (Spalte 5) sowie auf ihren Anteil an allen Behandlungstagen (Spalte 6) unterschiedlich aus.

### Rezidivierende depressive Störung (F33)

96,1 % der Patienten mit rezidivierenden depressiven Störungen hatten im Jahr 2024 mindestens einmal Kontakt zu einem Hausarzt (Tab. 4, Spalte 3) und 60,3 % waren aufgrund ihrer Erkrankung in hausärztlicher Behandlung (Tab. 4, Spalte 4). In der Gruppe der Patienten mit Kontakt zu einem Hausarzt entfielen 88 % der krankheitsspezifischen Behandlungstage (Spalte 5) und 50 % aller Behandlungstage (Spalte 6) auf die Hausärzte. 18,5 % der Patienten mit rezidivierenden depressiven Störungen waren aufgrund ihrer Erkrankung in psychiatrischer Behandlung. Auf die Psychiater entfielen 72 % der krankheitsspezifischen und 23 % aller Behandlungstage der psychiatrisch behandelten Patienten. Bei 17 % der Patienten erfolgte die Behandlung der Erkrankung durch nichtärztliche Psychotherapeuten. Auf sie entfielen 80 % der krankheitsspezifischen und 38 % aller Behandlungstage der psychotherapeutisch behandelten Patienten. Bei allen weiteren der 10 Fachgruppen mit dem höchsten Anteil krankheitsspezifischer Behandlungstage betrug der Anteil der durch sie krankheitsspezifisch behandelten Patienten maximal 8,1 % und der Anteil an den krankheitsspezifischen Behandlungstagen der Patienten maximal 68 %.

### Multiple Sklerose (MS; G35)

Die meisten Patienten mit MS wurden aufgrund ihrer Erkrankung von Hausärzten betreut (83 %). Auf die Hausärzte entfielen 60 % der krankheitsspezifischen Behandlungstage und 47 % aller Behandlungstage der MS-Patienten mit Hausarztkontakt. Bei 52 % der Patienten erfolgte die Behandlung der MS durch Neurologen. Auf Neurologen entfielen 42 % der krankheitsspezifischen und 29 % aller Behandlungstage der neurologisch behandelten Patienten. Bei 17 % der Patienten wurde MS von Nervenärzten^1^ behandelt. Auf sie entfielen 37 % der MS-spezifischen und 26 % aller Behandlungstage der nervenärztlich behandelten Patienten. Bei allen weiteren der 10 Fachgruppen mit dem höchsten Anteil krankheitsspezifischer Behandlungstage betrug der Anteil der durch sie behandelten Patienten maximal 10,4 % und der Anteil an den krankheitsspezifischen Behandlungstagen der Patienten maximal 36 %.

### Colitis ulcerosa (K51)

Patienten mit Colitis ulcerosa waren zu 84 % aufgrund ihrer Erkrankung bei einem Hausarzt, wobei 82 % der krankheitsspezifischen und 54 % aller Behandlungstage der hausärztlich betreuten Patienten auf die Hausärzte entfielen. Rund 33 % der Patienten mit Colitis ulcerosa waren aufgrund ihrer Erkrankung in gastroenterologischer Behandlung. In der Gruppe der gastroenterologisch behandelten Patienten entfielen 50 % der krankheitsspezifischen und 27 % aller Behandlungstage der Patienten auf diese Fachgruppe. Bei allen weiteren der 10 Fachgruppen mit dem höchsten Anteil krankheitsspezifischer Behandlungstage betrug der Anteil der durch sie behandelten Patienten maximal 3,8 % und der Anteil an den krankheitsspezifischen Behandlungstagen der Patienten maximal 49 %.

### Seropositive chronische Polyarthritis (M05)

Bei Patienten mit Polyarthritis konzentrierte sich das krankheitsspezifische Versorgungsgeschehen im Wesentlichen auf die Hausärzte und Rheumatologen, von denen jeweils 50 % der Patienten behandelt wurden. Wenn Patienten hausärztlich behandelt wurden, entfielen 88 % der krankheitsspezifischen und 52 % aller Behandlungstage der Patienten auf die Hausärzte. Der Behandlung durch Rheumatologen ließen sich 79 % der krankheitsspezifischen und 22 % aller Behandlungstage der Patienten mit Kontakt zu einem Rheumatologen zuordnen. 14 % der Patienten wurden aufgrund der Erkrankung von Orthopäden behandelt. Auf sie entfielen 76 % der krankheitsspezifischen, aber nur 15 % aller Behandlungstage der orthopädisch behandelten Patienten. Ein ähnlich hoher Anteil an den krankheitsspezifischen Behandlungstagen ist auch bei den durch Augenärzte behandelten Patienten zu erkennen (78 %). Allerdings ist der Anteil der von Augenärzten behandelten Patienten mit 0,9 % sehr gering. Bei allen weiteren der 10 Fachgruppen mit dem höchsten Anteil krankheitsspezifischer Behandlungstage betrug der Anteil der durch sie behandelten Patienten maximal 0,9 % und der Anteil an den krankheitsspezifischen Behandlungstagen der Patienten maximal 65 %.

### Krankheiten infolge Schädigung der tubulären Nierenfunktion (N25)

77 % der Patienten mit geschädigter Nierenfunktion wurden aufgrund ihrer Erkrankung durch Nephrologen behandelt. Dabei konnten 92 % der krankheitsspezifischen und 37 % aller Behandlungstage der nephrologisch versorgten Patienten dieser Fachgruppe zugeordnet werden. Zwar zeigte sich bei Gynäkologen ein ebenso hoher Anteil an den krankheitsspezifischen Behandlungstagen, aber der Anteil der durch sie behandelten Patienten und der Anteil an allen Behandlungstagen dieser Patienten war mit 0,4 % beziehungsweise 8 % sehr gering. 12 % der Patienten wurden durch sonstige Leistungserbringer (in der Regel Krankenhäuser, die für die ambulante Versorgung ermächtigt sind) versorgt, wobei 84 % der krankheitsspezifischen und 21 % aller Behandlungstage der durch sie behandelten Patienten auf diese entfielen. Lediglich 23 % der Patienten wurden aufgrund ihrer Erkrankung von Hausärzten behandelt, wobei ein hausärztlicher Anteil von 76 % an den krankheitsspezifischen und 43 % an allen Behandlungstagen hausärztlich versorgter Patienten bestand. Bei allen weiteren der 10 Fachgruppen mit dem höchsten Anteil krankheitsspezifischer Behandlungstage lagen deutlich niedrigere Anteile an behandelten Patienten (maximal 2 %) und ein maximaler Anteil an den krankheitsspezifischen Behandlungstagen von 81 % vor.

## Diskussion

Die Bundesregierung beabsichtigt, ein verbindliches Primärarztsystem durch Haus- und Kinderärzte einzuführen, wobei für Patienten mit „spezifischen schweren chronischen Erkrankungen“ geeignete (alternative) Lösungen erarbeitet werden sollen. Welche Erkrankungen unter die Kategorie „spezifische schwere chronische Erkrankungen“ fallen, ist derzeit noch unklar. Ziel der vorliegenden Arbeit war es daher, eine Methode darzustellen, nach der geeignete Erkrankungen ausgewählt und hinsichtlich des derzeitigen fachärztlichen Versorgungsanteils untersucht werden können. Aus 173 potenziell für eine fachärztliche Steuerung infrage kommenden ICD-3-Stellern mit hohem fachärztlichen Versorgungsanteil wurden exemplarisch 5 ICD-3-Steller ausgewählt.

Für diese zeigt sich, dass weiterhin der höchste Patientenanteil sowie die höchsten Anteile an Behandlungsfällen und Behandlungstagen insgesamt auf die Versorgung durch Hausärzte entfallen. Selbst bei Patienten mit chronischem Nierenversagen, bei denen aufgrund der Dialysebehandlung 83 % der Patienten regelmäßigen Kontakt zum Nephrologen haben und die Nephrologen mit einem Patientenanteil von 77 % den höchsten Anteil an der Behandlung des Nierenversagens haben, entfallen auf die Nephrologen im Mittel nur rund 37 % aller Behandlungstage der Patienten, für die auch eine Diagnose aus der Kategorie N25 codiert wurde. Geht man rein nach diesen Mittelwerten, so dürften sich Nephrologen sehr gut überlegen, ob sie als Bezugspraxis für einen Patienten auch die Koordination der Behandlung der vorliegenden Komorbiditäten insgesamt übernehmen wollen. Das Muster ist für andere Fachrichtungen für die 5 ausgewählten Indikationsbereiche vergleichbar. Selbst bei hoher Bedeutung für die fachspezifischen Behandlungen ist der Anteil jeder einzelnen Fachrichtung an den insgesamt anfallenden Behandlungstagen der hausärztlichen Versorgung deutlich nachgeordnet. Der Zusatzaufwand einer Koordination fachfremder Behandlungen dürfte erheblich sein und daher nur in noch spezifischer abgegrenzten Patientensubgruppen oder für bestimmte Behandlungsepisoden infrage kommen. Dies könnte im Fall des chronischen Nierenversagens für einige Patienten etwa in der terminalen Stufe 5 infrage kommen, in der Patienten im Mittel mehr Behandlungstage beim Nephrologen als beim Hausarzt aufweisen und auch Impfungen zu rund einem Drittel etwa von Nephrologen durchgeführt werden, wie wir in einer punktuellen Sonderauswertung überprüft haben (Ergebnisse können bei den Autoren erfragt werden). Bei Patienten mit onkologischen Erkrankungen ist zudem aufgrund der episodenbezogenen fachärztlichen Versorgung eine vorübergehend höhere Facharztbindung zu erkennen [4].

### Limitationen

Das beschriebene Vorgehen ist mit einigen Limitationen verbunden. So wurden „Behandlungstage“ als Surrogat für Arzt-Patienten-Kontakte verwendet. Dabei können Behandlungstage in Fällen, in denen mehr als eine Diagnose codiert oder mehrere Fachrichtungen in einem Behandlungsfall zusammengefasst sind, nicht eindeutig einer Diagnose oder Fachgruppe zugeordnet werden. In diesen Fällen wurden die Behandlungstage allen enthaltenen Diagnosen und Fachgruppen zugeordnet, was zu einer Über- oder Unterschätzung der krankheitsspezifischen Behandlungstage auf der Ebene der Fachgruppen geführt haben kann. Darüber hinaus handelt es sich bei „Behandlungstagen“ eigentlich um Abrechnungstage. Das heißt, nur wenn bei erneutem Kontakt innerhalb eines Behandlungsfalles die Möglichkeit der Abrechnung einer neuen Gebührenordnungsposition (GOP) besteht, wird ein erneutes Abrechnungsdatum mit einem weiteren Behandlungstag generiert. Insbesondere bei Hausärzten, bei denen viele Einzelleistungen in den Quartalspauschalen enthalten sind und nach dem ersten Kontakt innerhalb des Quartals häufig keine weiteren GOP abgerechnet werden können, wird daher die tatsächliche Anzahl an Arzt-Patienten-Kontakten durch die Behandlungstage unterschätzt. Eine weitere Limitation betrifft die Abrechnungsdiagnosen. Bei den in den Abrechnungsdaten enthaltenen Diagnosen handelt es sich um abrechnungs- und therapiebegründende Diagnosen. So kann allein die erneute Verordnung eines Arzneimittels zur Angabe einer Diagnose führen, ohne dass eine weitere ärztliche Behandlung erfolgt ist. Dies kann zu einer Überschätzung der krankheitsspezifischen Behandlungstage geführt haben. Weiterhin basierte der für die Identifikation geeigneter chronischer Erkrankungen gewählte methodische Ansatz auf ICD-3-Stellern. ICD-3-Steller sind allerdings nicht durchweg geeignet, chronische Erkrankungen vollständig zu erfassen, da diese mitunter durch mehrere ICD-3-Steller charakterisiert sein können. Die 5 untersuchten ICD-3-Steller wurden so ausgewählt, dass diese Limitation hier vernachlässigt werden kann. Die Auswahl der näher betrachteten ICD-3-Steller aus dem Pool der ICD-3-Steller mit hohem fachärztlichen Versorgungsanteil erfolgte allerdings nur exemplarisch. Es ist möglich, dass unter den übrigen nicht näher betrachteten Codes Beispiele für überwiegend durch eine Fachgruppe behandelte chronische Erkrankungen zu finden sind.

## Fazit

Die vorliegende Arbeit legt daher nahe, dass der Spielraum für eine sachgerechte Formulierung von Ausnahmen durch den Gesetz- oder Verordnungsgeber eng begrenzt sein dürfte. Auch in der Literatur wird die Übernahme der Koordinationsfunktion für die gesamte Versorgung von Patienten durch spezialisierte Fachärzte eher als Defizit eines funktionierenden Primärarztsystems verstanden [5, 6]. Davon könnten definierte Episoden ausgenommen sein. Allerdings zeigen andere Literaturstellen die Schwierigkeiten sowohl bei der Definition der Rolle einer fachübergreifenden Versorgungskoordination durch spezialisierte Fachärzte in einem Primärarztsystem als auch bei der Bereitschaft der Fachärzte, eine solche Rolle zu übernehmen [7]. Auch etwa für seltene Erkrankungen, die auch in anderen Primärarztsystemen eher durch spezifische fachärztlich ausgerichtete Ambulanzen behandelt werden, finden sich kaum Beispiele einer fachärztlich koordinierten Versorgung [8]. Die Formulierung „geeigneter (alternativer) Lösungen für Patienten mit spezifischen schweren chronischen Erkrankungen“ sollte daher eher einen Möglichkeitenraum beschreiben, der im Einzelfall in Aufgabenabstimmung und zeitlicher Dimension zwischen Facharzt- und Primärarztpraxis ausgefüllt werden kann und in einer qualifizierten Überweisung dokumentiert wird.

## Data Availability

Die der Studie zugrunde liegenden Daten sind als Gesundheits- und Sozialdaten aufgrund der Datenschutzvorgaben des SGB V, des SGB X und der DSGVO nicht öffentlich zugänglich. Die in Tab. 2 auszugsweise dargestellten aggregierten Daten sowie die aggregierten Daten, die der Auswahl der ICD-3-Steller in Tab. 3 zugrunde liegen, sind auf begründete Anfrage bei der Korrespondenzperson erhältlich.

## Footnotes

1 Die Bezeichnungen der Fachgruppen folgen der Abrechnungssystematik des Einheitlichen Bewertungsmaßstabs. Neurologie und Nervenheilkunde werden darin als getrennte Fachgruppen ausgewiesen.
